# Use of Nanotechnology to Mitigate Biofouling in Stainless Steel Devices Used in Food Processing, Healthcare, and Marine Environments

**DOI:** 10.3390/toxics10010035

**Published:** 2022-01-12

**Authors:** Hugo Pérez, Gregorio Vargas, Rodolfo Silva

**Affiliations:** 1 Sustentabilidad de los Recursos Naturales y Energía, Centro de Investigación y de Estudios Avanzados del Instituto Politécnico Nacional (CINVESTAV) Unidad Saltillo, Ramos Arizpe 25900, Mexico; hugo.perez@cinvestav.mx; 2 Instituto de Ingeniería, Universidad Nacional Autónoma de México, Mexico City 04510, Mexico; RSilvaC@iingen.unam.mx

**Keywords:** nanotechnology, biofouling, stainless steel, marine environments, food processing, health care

## Abstract

In humid environments, the formation of biofilms and microfouling are known to be the detrimental processes that first occur on stainless steel surfaces. This is known as biofouling. Subsequently, the conditions created by metabolites and the activity of organisms trigger corrosion of the metal and accelerate corrosion locally, causing a deterioration in, and alterations to, the performance of devices made of stainless steel. The microorganisms which thus affect stainless steel are mainly algae and bacteria. Within the macroorganisms that then damage the steel, mollusks and crustaceans are the most commonly observed. The aim of this review was to identify the mechanisms involved in biofouling on stainless steel and to evaluate the research done on preventing or mitigating this problem using nanotechnology in humid environments in three areas of human activity: food manufacturing, the implantation of medical devices, and infrastructure in marine settings. Of these protective processes that modify the steel surfaces, three approaches were examined: the use of inorganic nanoparticles; the use of polymeric coatings; and, finally, the generation of nanotextures.

## 1. Introduction

When a biofilm forms on a surface, and is subsequently colonized by micro and macroorganisms, this is known as biofouling, and it can lead to the deterioration of the surface. It is a process that occurs naturally in many areas of human activity, including marine structures, food processing, and in the use of medical implants and devices [1,2]. The negative effects of biofouling on the performance and maintenance of stainless steel surface components may reduce their lifetime. 

Stainless steels are widely used for their anti-corrosion properties in different wet environments. However, in the presence of electrolytes, stainless steels are susceptible to the formation of biofilms and consequently to biofouling and microbiologically induced corrosion. There are three main areas where increasing the useful life of stainless steel components would be beneficial: for components placed in seawater, those used in food processing, and in biomedical devices [3,4,5].

Stainless steel is resistant to corrosion due to the passive chromium oxide layer that forms on its surface. However, in certain operating conditions, microorganisms can colonize the surface, [6,7], accelerating corrosion reactions, and/or changing the corrosion mechanisms. This effect is commonly referred to as “microbiologically influenced corrosion” (MIC) [6].

The US National Association of Corrosion Engineers (NACE) [8] estimates that annual losses associated with the corrosion of consumer and industrial goods make up around 2–4% of the GDP of all nations. Other estimates indicate that microbial processes are directly, or indirectly, responsible for about 30% of these losses [9].

To address the problems caused by biofouling on stainless steel surfaces, this paper reviews the mechanism of biofilm formation in three different environments: seawater, body fluids (blood, saliva, and urine), and food processing. Depending on the environment in which metal surfaces are exposed, different technologies have been developed to mitigate the problem of biofouling. Most of the alternatives explored have focused on inhibiting biofilm formation. 

It was decided to focus on nanotechnology for the following reasons:

- A large surface area is available for the interaction of nanoparticles with the cells of microorganisms [10,11,12];
- Due to the smaller size of nanoparticles, they are easier to transport into the cells of microorganisms, which facilitates their elimination or the inhibition of their development [10,11,12];
- The wide spectrum of nanoparticles available with different mechanisms of biocidal action allows them to be used synergistically to inhibit the formation of biofilms and consequently avoid micro and macro fouling [13];
- The controlled release capacity of “smart”/stimuli-responsive nanomaterials. Mesoporous silica nanocapsules, layered double hydroxides, halloysite nanotubes, and surface functionalization can increase antifouling activity time by up to one year through the controlled release of biocides. Controlled release can also reduce the toxicity of biocides relative to their application in free form. Furthermore, it was demonstrated that some of these “smart” nanomaterials exhibit eco-friendly properties since the controlled release capacity ensures a significant reduction in toxicity and environmental hazards compared with the conventional booster biocides [14,15].

## 2. Environments in Which Biofouling Often Occurs

**Figure 1:**
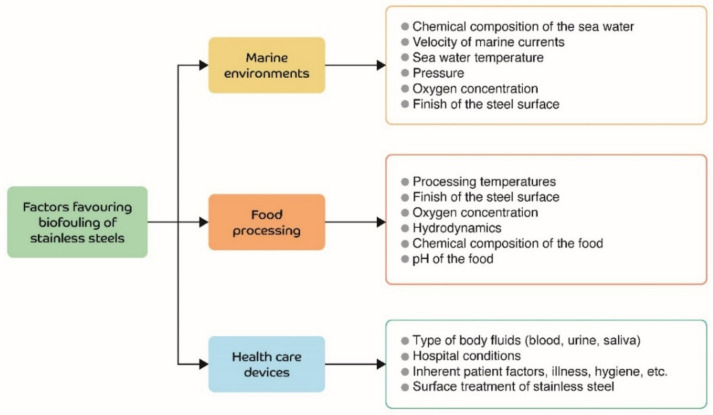
Biofouling factors in stainless steels devices used in the environments discussed.

Stainless steel is widely used in marine environments, food processing, and in biomedical implants and devices. Figure 1 shows the most important factors influencing the biofouling of stainless steel surfaces in these three environments. Each of these factors is briefly explained below.

### 2.1. Marine Environments

At the interfaces between interacting bodies and/or between surfaces and their environment, deterioration may occur. This degradation may be purely mechanical, such as erosion and abrasion, or may involve significant chemical aspects, such as corrosion of a metal, or tribocorrosion (chemical and mechanical processes occurring simultaneously). Corrosion, therefore, cannot be defined without reference to the characteristics of the material and the environment in which it is found.

The oceans are environments that enable the creation of life and the conversion of energy, but they are also extremely aggressive environments. Seawater is a complex mixture of various salts, dissolved gases, trace elements, suspended solids, decomposed organic matter, and living organisms [16]. In seawater, the behaviour of metals is linked to oxygen content, the velocity of currents, temperature, pollution, marine organisms, and the position of the materials with respect to the mean sea tide [17,18]. 

**Figure 2:**
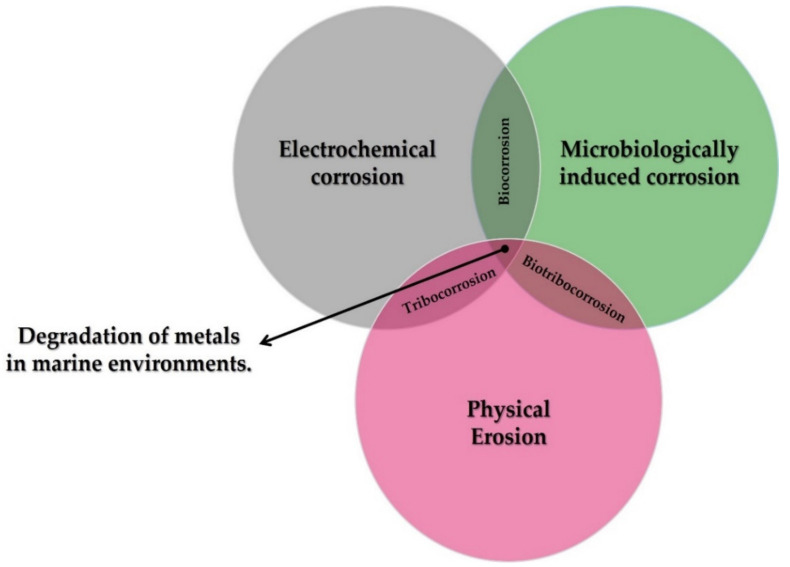
Different forms of metal degradation in marine environments.

The contribution of each degradation mechanism, both individually and in combination with each other, in marine environments can be seen in Figure 2. 

According to Little and De Palma [19], the factors that affect the rate of biofouling are: the chemical composition of the water, marine current flow, pressure, shear stress, physical and chemical characteristics of the substrate, the photosynthetic activity of aquatic plants, changes in salinity, seasonal changes, turbidity, oxygen levels, and the depth of the metal surface relative to sea level. 

**Seawater chemistry** [19]. Nutrient-rich waters produce biofilms at a rapid rate, while waters deficient in nutrients produce biofilms more slowly. The attachment of microorganisms to the surface of substrates is closely related to the production of the organic material used to anchor microorganisms. In carbon-rich and nitrogen-deficient waters, microbes tend to produce large amounts of organic material. This material also stimulates the colonization of other species and the concentration of nutrients. Consequently, one of the most critical factors governing the distribution of bacteria in the sea is the availability of nutrients.

**Velocity of marine currents**. Relini et al. [20] reported that ascidians and bryozoans might be prevented from adhering to surfaces at velocities above 0.4 m/s. Huve [21] reported that barnacle larvae might be prevented from settling on submerged plates at velocities of over 1.0 m/s. This parameter offers an opportunity to find out about the biofouling process; these values could be considered critical in the design stage of this type of antifouling method. 

**Sea temperature**. This controls the adhesion and growth rate of microorganisms on steel surfaces; the growth rate increases as the temperature increases. In colder waters, the reproduction of microorganisms begins in spring and continues throughout the summer. Whereas, in tropical waters, fouling biota reproduce vigorously almost all year round [19].

**Pressure**. Jannasch et al. [22] found reduced microbial activity in the deep sea and attributed this to the combined effects of temperature and hydrostatic pressure. Berger [23] observed slow microbial activity in the deep ocean due to the inhibitory effects of pressure on the biosynthesis process. 

**Shear stress** [22]. The growth of biofouling can be controlled in turbulent flows that generate shear stresses. It was observed that significant biofouling cannot occur when the shear stress exceeds 200 g/cm^2^ (19,613.3 Pa).

**Surface treatment of stainless steel** [19]. Polished surfaces are initially more resistant to biofouling than rough surfaces; however, the effect is quickly overcome as microorganism growth occurs. Cracks in bolted joints or welds without complete penetration are preferred sites for the colonization of microorganisms. For example, barnacle cyprinids settle in depressions, while algal spores prefer shallow crevices.

### 2.2. Food Processing 

Factors that have a significant impact on the biofouling of steel surfaces in the food processing industry [24,25,26] include: the substrate surface finish, processing temperatures, oxygen concentrations, pH, and the nature and chemical composition of the food being processed. 

**Surface treatment of stainless steel**. The roughness of the surface affects the start of biofilm formation process, providing sites where microorganisms can lodge and protect themselves from external elements [27,28].

Materials with hydrophobic surfaces favour the adhesion of bacteria with hydrophobic properties while hydrophilic surfaces favour the adhesion of hydrophilic bacteria [29,30]. Opposite electrostatic charges between the substrate surface and bacterial cells are also attractive sites for the adhesion of microorganisms [31].

**Processing temperature**. Lower temperatures result in more uniform properties in polysaccharides, stimulating biofilm formation [32,33]. In addition, lower temperatures decrease the hydrophobicity of the cell surface, leading to less biofilm formation [34]. On the other hand, high temperatures can compromise the life of microorganisms [25]. Temperature also affects the viscosity of polysaccharides and the solubility of food components, causing the precipitation of compounds that favour biofilm formation [25,35].

**Oxygen concentration**. A decrease in oxygen concentration within the biofilm reduces bacterial metabolic activity and inhibits bacterial growth [36]. In general, an increase of oxygen on the surface causes a decrease in the hydrophobicity of the system [37].

**Hydrodynamics of the process**. In turbulent flow, macromolecules, nutrients and the transport velocity of microorganisms can also affect the biofilm formation process [38].

**Chemical composition of food**. Food properties influence bacterial adhesion by changing the surface characteristics of both the bacteria and substrates, favouring the formation of polysaccharides in the biofilm [30]. In addition, the composition and concentration of the food matrix causes changes in bacterial physiology, consequently affecting the adhesion of the biofilm on the substrate [25].

**pH**. Increased acidic or alkaline conditions modify the physicochemical properties of cell walls, as well as bacterial gene expression (quorum sensing system), which directly impacts bacterial adhesion. The level of impact of this feature will depend on the type of bacteria [25].

### 2.3. Devices Used in Human Health Care

The main factors in this area which affect the biofouling process are: the type of body fluid, the surface finish of the steel, nosocomial sanitary conditions and factors inherent to the recipient patient. 

**Type of body fluid**. When a device is implanted, it is immediately surrounded by body fluids such as blood, urine, saliva or mucus, which have unique distinguishing characteristics, such as pH or chemical composition, that can influence bacterial adhesion or even corrode the device and generate cavities where these microorganisms can settle [39].

**Surface treatment of stainless steel**. This has a similar influence as that indicated for food processing (rough surfaces favour biofilm formation). For example, *Staphylococcus epidermidis* cells readily adhere to the surface of a device due to factors such as surface tension, hydrophilicity and electrostatic forces [39].

**Hospital conditions**. Contaminated infusions, unhygienic practices of healthcare staff and the skin of the actual patient are sources of biofilm-forming microorganisms on catheters [39]. For example, microorganisms gain access to the catheter by migrating externally from the skin along the external surface of the catheter, or internally from the catheter hub or pole, rapidly colonizing its surfaces.

**Factors inherent in the patient**. *Staphylococcus aureus* adhesion seems to be more dependent on the presence of host tissue ligands, such as fibronectin, fibrinogen, and collagen (bacterial hosts), which may promote adhesion of microorganisms [39].

## 3. The Biofouling Process

In its initial stages, the biofouling process is governed by physicochemical forces such as electron transfer, Brownian motion, electrostatic interactions, and Van der Waals forces [2]. The stages in the biofouling process in seawater devices are described below.

### 3.1. Stage 1. Biofilm Formation

One minute after immersion of the component, adhesion of organic compounds (proteins and polysaccharides) will have occurred. These proteins and polysaccharides come from the existing nutrients in seawater and are formed from nitrogen and carbon from biological cycles (excretions and the death of marine organisms). These nutrients in turn attract the first colonizing organisms forming a biofilm. A biofilm is a set of immobilized cells embedded in a dense and complex extracellular polymeric matrix produced by the microorganisms themselves, on a substrate [1]. Biofouling is a very complex phenomenon that is not yet fully understood, formed through mechanisms such as crystallization, particle contamination, chemical precipitation, corrosion, and solidification [40]. The microorganisms that may be involved in marine biofouling are mainly the sticky, or sessile, forms present in shallower waters along the coast [2]. Zhang et al. [41] identified more than 7300 biofilm forming species in the sea, of these it is as yet uncertain how many can adapt to the conditions on human infrastructure and adhere to them. These organisms have also adapted to fluctuations in environmental conditions such as temperature, ocean current flow, and salinity [2,42].

The metabolic activity of the cells within the biofilm leads to a reduction in the oxygen concentration on the metal surface, producing a differential aeration cell underneath the biofilm, and generating an anode. On the other hand, in the uncolonized areas exposed to maximum oxygen concentrations, a cathode is generated. The basic corrosion mechanism involves the flow of electrons from an anode to a cathode region, where the electron acceptor is oxygen [7].

The biofilm is made up of a cell mass that represents only 2–5% of the total weight, the rest is made up of an extracellular polymeric substance. This substance comprises various polysaccharides, proteins, nucleic acids, glycoproteins, phospholipids, water, and other surfactants. These polymers are mainly polysaccharide fibrils based on glucose and fructose [43]. The proportion of excreted extracellular substances depends on the type of microorganism [44].

Both the extracellular adhesive substances and the roughness of the steel surface help to trap more particles and organisms [2], protecting them against environmental stresses, including: desiccation; changes in temperature, pH predators, and toxins (in 10 to 1000 times higher concentrations); UV exposure; and facilitating the capture of necessary nutrients (thanks to the polymer gel matrix in which they are embedded) [45]. This access to nutrients and organic molecules is probably the main advantage that bacteria within biofilms have [3,46,47]. Once established on the metal, the marine biofilm rapidly colonizes surfaces such as stainless steel, accelerating their corrosion [48].

### 3.2. Stage 2. Primary Colonization

The colony formation of microorganisms as bacteria or algae occurs during the first 24 h. At this stage the biofilm becomes a more complex community that generally includes multicellular organisms, herbivores, and decaying organisms. Bacterial adhesion occurs through interactions with planktonic cells, such as electrostatic interactions, gravity, and water flow. After the initial reversible adsorption, bacteria use extracellular polymers to temporarily attach themselves to the surface.

Some studies suggest there is a significant difference in biofilm formation depending on whether it occurs in marine environments, or in hospitals and food processing machines [49,50,51,52]. Cell-to-cell communication plays an essential role in the synchronization of processes within the biofilm and is carried out through the quorum sensing system (QS). This system regulates gene expression in response to cell population density. This communication process is carried out by compounds called “autoinducers”, which serve as a chemical signal to induce gene expression in the cell collective [53].

Gram-positive and gram-negative bacteria use quorum sensing communication circuits to regulate a wide variety of physiological activities, such as symbiosis, virulence, competition, conjugation, antibiotic production, motility, sporulation, etc. In general, gram-negative bacteria use acyl-homoserine lactone as an autoinducer, whereas gram-positive bacteria use processed oligopeptides as autoinducers [53]. The quorum sensing communication circuit organizes the microorganisms inside the biofilm and helps it to mature.

In medical devices [54,55], bacteria can adhere to the metal surfaces, causing severe diseases in patients when they mature [2,54]. Moreover, as the biofilm blocks the patient’s defence mechanisms, the action of antibiotics and other chemical agents is inhibited [31,32]. Body fluids can also cause corrosion of a steel device, releasing chromium and nickel ions, which can accumulate in the tissues and put the patient’s health at risk [56].

### 3.3. Stage 3. Secondary Colonization (Micro Encrustation)

#### 3.3.1. Marine Environments

The adhesion of microalgae and protozoa spores occurs during the first week. Colonization by microorganisms in the sea begins after about 1 h. Both bacteria and algae can attach to marine structures such as ships, pipelines, and heat exchangers [2], reducing operational efficiency and increasing maintenance costs [57]. Dang et al. [46] and Dang and Lovell [57] found that bacteria colonize metal surfaces found in the sea, and that their cells can determine the structure and function of a mature biofilm. Railkin et al. [58] have determined that the observed ratio in marine biofilms of bacteria, diatoms and flagellates is 640:4:1. Some species of molluscs and crustaceans also colonize such surfaces, which leads to a considerable increase in greenhouse gas emissions [59].

Colonization of unicellular eukaryotes (e.g., diatoms, yeasts, and protozoa) usually begins several days after immersion [46]. Later, colonization by multicellular eukaryotes occurs. The main eukaryotic microorganisms are diatoms, fungi, and protozoa, with diatoms being the dominant microorganisms. Diatom adhesion is a more complicated process than bacterial adhesion, since most diatoms lack flagella, so they cannot actively approach a specific surface, but passively precipitate on the substrate. Benthic diatoms approach surfaces through gravitational effects or ocean currents. Planktonic diatoms, which have almost the same specific gravity as seawater, precipitate on surfaces through turbulence mainly. In addition, electrostatic interactions such as Coulomb attraction and electrostatic contact potential are involved [59,60].

After the diatoms precipitate on the steel surface, they form a reversible bond called primary adhesion through the secretion of extracellular polymeric substances. Subsequently, they reorient themselves and move across the surface to positions more suited to their preferences, a process called diatom gliding. The extracellular polymeric substance of diatoms is composed of sulphated carboxylic acid polysaccharides, which are involved in primary adhesion, and proteoglycans, which are involved in diatom gliding and stabilizing the cross-linking of the biofilm matrix [60].

To mitigate the action of microorganisms, it is essential to specify which ones are present, as well as their growth mechanisms. Among the bacteria are: sulphate reducing bacteria (SRB) [61], acid producing bacteria (APB) [62], manganese reducing bacteria (MRBn) [61,62], and iron oxidizing bacteria (IOB) [61,63], which generally settle in the biofilms formed on the surface of the steel.

Figueroa de Gil et al. [64] studied the effect of SRB (Desulfotomaculum thermoacetoxidians) on the corrosion of 316L stainless steel; the combined effect of oxygen and sulphide ions on the passive film of the steel, produced localized corrosion on the steel surface. This effect is still greater if hydrogen sulphide (H_2_S) is formed, as this reduces the pH in the affected zone, leading to pitting on the steel surface. A similar effect was also described by Santander Morales et al. [65] with the bacterium *Desulfovibrio desulfuricans*. They too observed the colonization of the surface of 316L stainless steel and pitting.

Emerson et al. [66] made an analysis of Fe-oxidizing bacteria (FeOB), explaining that this type of bacteria is oxygen-dependent, develops in neutral pH and belongs to the group of proteobacteria. Acting under aerobic conditions they consume oxygen, leaving conditions conducive for SRB to act under anaerobic conditions. FeOB also provide iron and sulphide ions, which is important for the pitting mechanism mentioned above by promoting the presence of anodic sites.

In marine environments, the dominant species is *Mariprofundus ferroxydans*. This is the most documented bacterium; it does not use sulphur, hydrogen, and ammonium compounds, nor organic substrates for its growth. It is a mesophilic microorganism with a growth limit temperature of over 30 °C, with genes to perform autotrophy. *Marinobacter aquaeolei*, some pseudoalteromonas and pseudomonas bacteria were also identified. 

#### 3.3.2. Food Processing

Microorganisms can adhere to equipment, pipes, conveyor systems, and tanks in the food industry [25,27], reducing heat transfer, generating energy losses, increasing the frictional resistance of fluids, and accelerating the corrosion process [33,34]. In addition, their adhesion can cause pathogenic diseases transmitted through contaminated food [24,25].

The environmental conditions in the food processing industry favour the proliferation of various types of microbes that can form biofilms, such as the bacteria *Listeria monocytogenes*, *Salmonella enterica*, *Escherichia coli*, or *Pseudomonas aeruginosa* and *Staphylococcus aureus* [24,26].

#### 3.3.3. Implants and Biomedical Devices

In prostheses and in medical implants, such as pacemakers, insulin pumps, operating room monitors, and defibrillators (coronary stents, valves and catheters are those most referred to in the literature) colonization will depend on the type of device in question. Biofilm formed on a urinary catheter may contain *Staphylococcus epidermidis*, *Enterococcus faecalis*, *Escherichia coli*, *Proteus mirabilis*, *Pseudomonas aeruginosa*, *Klebsiella pneumoniae,* and other gram-negative bacteria [67]. The biofilm formed on a central venous catheter may contain *Staphylococcus epidermidis*, *Staphylococcus aureus*, *Candida albicans*, *Pseudomonas aeruginosa*, *Klebsiella pneumoniae*, and *Enterococcus faecalis* bacteria. All these bacteria commonly originate from the patient’s skin, medical device, or healthcare personnel [68,69]. Both *Staphylococcus aureus* and *Staphylococcus epidermidis* are estimated to cause about 40–50% of prosthetic heart valve infections, 50–70% of catheter biofilm infections, and 87% of bloodstream infections [68].

### 3.4. Stage 4. Tertiary Colonization (Macro Encrustation)

During the second and third week, occurs the adhesion of organisms as larvae, crustaceans, cnidarians, molluscs, polychaetes, and tunicates. Macroorganisms such as algae, spores, barnacles, cnidarians, marine fungi and protozoa, polychaetes, tunicates, coelenterates, and other molluscs, as well as spores, can be attracted by sensory stimuli or can be trapped by the polymeric matrix of the biofilm. This is known as macrofouling [43,70]. The macroorganisms settle on the surface of stainless steel and this is the most noticeable and disturbing phase of biofouling, increasing the weight of the structure. It can only happen after microfouling [43,70]. Typically, macrofouling species have rapid metamorphosis and growth rates, a low degree of substrate preference, and adapt well to different environments.

The settlement and growth of marine microorganisms involve invertebrates, such as mussels and barnacles, along with the growth of macroalgae [43,48]. Some of the most common macrofouling organisms are shown in Table 1. 

## 4. Use of Nanotechnology to Mitigate the Biofouling Process on Stainless Steel

To mitigate biofilm formation and the adhesion of microorganisms on steel surfaces in seawater, antifouling paints based on copper or tin compounds have been used. According to Gipperth [83], tributyl tin (TBT) has been used since the 1970s due to its long shelf life, and has is very effective against the microorganisms mentioned. These compounds were banned in 2008 [2] as they cause abnormal development in non-target marine biota, such as oysters and mussels. The banning of paints made from tributyl tin (TBT) has brought about a significant change in antifouling coatings for marine structures and components.

Table 2 shows the microorganisms whose adhesion was inhibited using nanotechnology. Significant inhibitory effects were observed for 18 different bacteria (17 reported in marine environments, four in food processing and five in health care). Adhesion was also inhibited in five types of algae in marine environments.

The bacteria most studied were *Staphylococcus aureus* (gram-positive) and *Escherichia coli* (gram-negative); they are the most common cause of disease and infection in humans [93,94]. Analysis of them could be used as an inhibition model for gram-positive and gram-negative bacteria. Zotolla and Sasahara [95] reported that biofilm formation occurs when the number of adherent cells is 10^6^ to 10^7^ CFU/cm^2^. 

Three lines of research on the use of nanotechnology to reduce biofouling of stainless-steel surfaces were identified:

- the use of metallic nanoparticles in organic matrix coatings;
- the generation of nano textures on the surface.

The inhibition mechanisms generated by these modifications on stainless steel are described below.

### 4.1. Use of Metallic Nanoparticles in Organic Matrix Coatings

The use of nanoparticles to inhibit biofilm formation on stainless steel surfaces has been reported, killing up to 18 types of bacteria [44,85,87] and five types of algae [86,87,91]. The main difficulties in this are the homogeneous distribution of nanoparticles on the substrate surface and the controlled release of active ions. An advantage of the use of nanoparticles is the large surface area available for interaction with the microorganisms, becoming more cytotoxic to them [11,12]. This is possible as the particle is much smaller than the main compounds that form the structure of the cell of the microorganisms, making the interaction easy. Four nanoparticles used in treatments to reduce biofouling are described below. 

**Figure 3:**
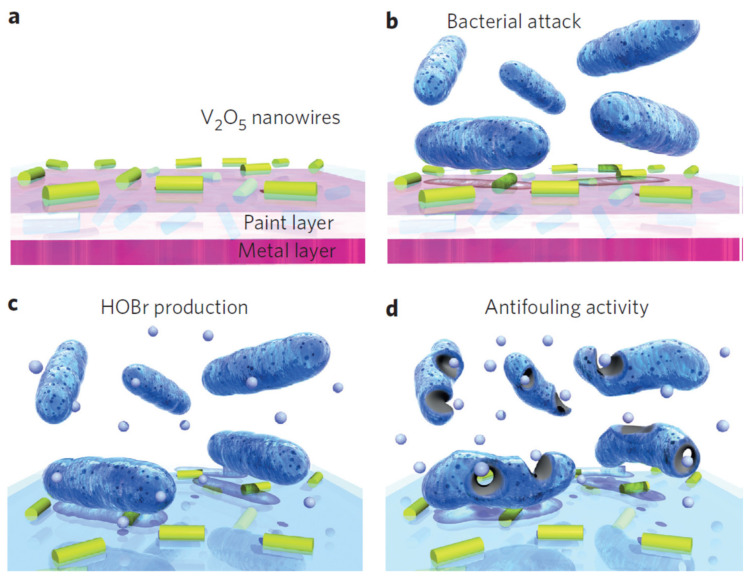
Bactericidal properties of V_2_O_5_ nanowires mixed with antifouling paint proposed by Natalio et al. (Reproduced from [84], published by Springer Nature, 2012.). (**a**) Nanoparticles (yellow–green rods) are embedded in a matrix (paint) and applied onto a metal surface. (**b**) They can be attacked easily by bacteria. (**c**) The V_2_O_5_/paint nanocomposite displays an intrinsic biomimetic catalytic activity, as found in vanadium haloperoxidases (V-HPOs); that is, in the presence of substrates such as Br_2_ and H_2_O_2_, small amounts of hypobromous acid (HOBr, small light blue spheres) are produced continuously. (**d**) The released HOBr interferes with the quorum sensing system of bacteria, preventing adhesion of the bacteria and biofilm formation.

**Vanadium**. Natalio et al. [84] developed vanadium pentoxide (V_2_O_5_) nanowires (300 nm long × 20 nm wide), which showed inhibitory activity against bacteria *Staphylococcus aureus* (96% reduction) and *Escherichia coli* (78% reduction), without affecting marine biota. They also demonstrated that the activity of the nanowires is stable for several catalytic, or duty, cycles. The biocidal mechanism of V_2_O_5_ nanowires was explained by their bromination activity, such as the functioning of vanadium haloperoxidase (V-HPO) enzymes, which produce hypobromous acid (HBrO) at pH 8–8.3 (see Figure 3). The nanowires catalyze the oxidation of bromide ions (Br^−^) to HBrO in the presence of hydrogen peroxide (H_2_O_2_), forming a reactive oxygen molecule which exerts vigorous antibacterial activity and interferes with the bacterium quorum sensing system. 

These authors evaluated the impact of V_2_O_5_ nanowires on *Artemia franciscana* instar II-III larvae and found them to be 14 to 1000 times less toxic on non-target species than zinc and copper nanoparticles, respectively. These nanoparticles are therefore a possible alternative to the antifouling products currently available commercially.

**Figure 4:**
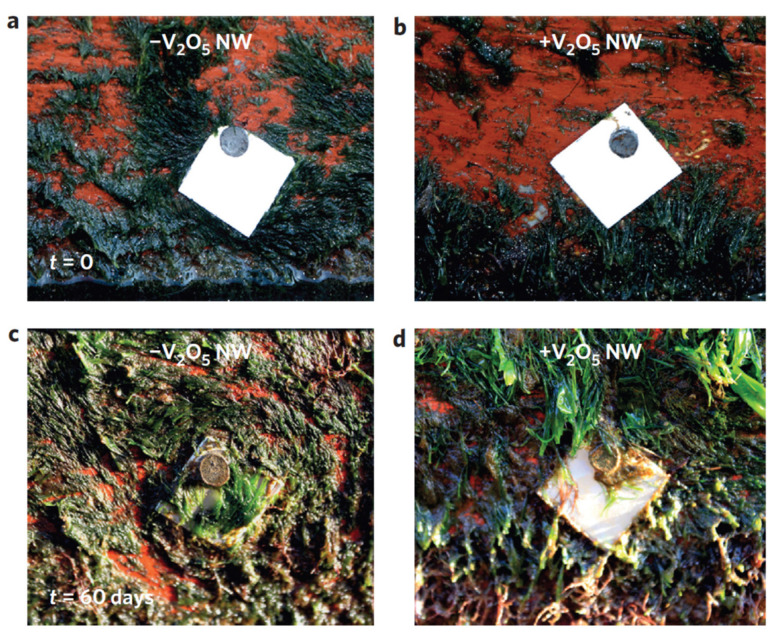
Effect of nanowires on biofouling in situ observed by Natalio et al. (Reproduced from [84], published by Springer Nature, 2012.) Digital image of a stainless steel plate (2 × 2 cm) covered with a commercially available paint for boat hulls without (−V_2_O_5_ nw) and with (+V_2_O_5_ nw) V_2_O_5_ nanowires. The plates were fixed to a boat hull. (**a**,**b**) Immediately afterwards, both stainless steel plates (with and without V_2_O_5_ nanowires) had clean surfaces. The boat was kept in seawater (lagoon with tidal water directly connected to the Atlantic Ocean). After 60 days, the boat was taken from the water; (**c**) The painted stainless-steel plates with no V_2_O_5_ nanowires showed severe natural biofouling; (**d**) The plates with the V_2_O_5_ nanowires had no biofouling whatsoever.

With the addition of nanowires to a commercial antifouling paint, no biofouling occurred on the stainless steel surface in a 60-day test (Figure 4). The inhibitory mechanism of this combined approach, involves, first, an attack of the bacteria on the nanowires, which in the presence of Br_2_ and H_2_O_2_, continuously produces HBrO (increasing the pH locally) and reactive oxygen molecules. Both these actions interfere with the quorum sensing system of the bacteria, preventing their adhesion and the formation of a biofilm [84]. It was suggested that hypobromous acid and reactive oxygen interfere with peptide bonding (amide-like bonding between −NH_2_ and COOH termini on two amino acids). This action prevents the emission of the autoinductor. The autoinducer in gram-positive bacteria is usually a modified peptide, containing 7–9 amino acids in length, and a characteristic thiolactone ring [96]. In gram-negative bacteria, the autoinducers are acyl homoserine lactone (Al-1) and furanosyl borate diester [97].

**Figure 5:**
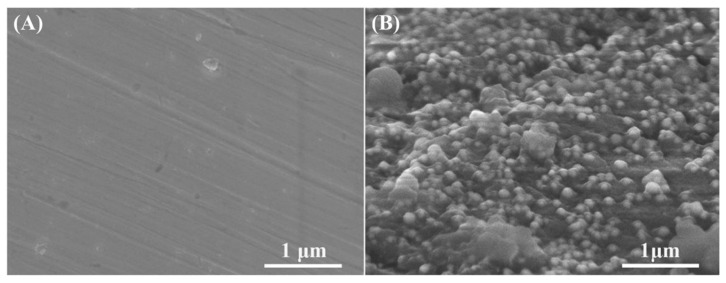
Field emission scanning electron microscope (FESEM) images of: (**A**) untreated; and (**B**) copper nanoparticles (Cu NPs) surface. Figure derived from the work of Cao et al. (Reproduced from [86], published by John Wiley and Sons, 2019.).

**Copper**. Cao et al. [86] were able to reduce the adhesion of *Escherichia coli* and *Staphylococcus aureus* bacteria by 92.1% and 80.4%, respectively. They also observed an inhibition of the diatomaceous alga *Phaeodactylum tricornutum* by 98.15%. To achieve this, they coated 304 stainless steel with polydopamine and copper aggregates. The polydopamine provides sufficient adhesion and permits a homogeneous distribution of the copper nanoparticles on the stainless steel surface (see Figure 5), due to their reaction with the amino and hydroxyl groups of the polydopamine.

**Figure 6:**
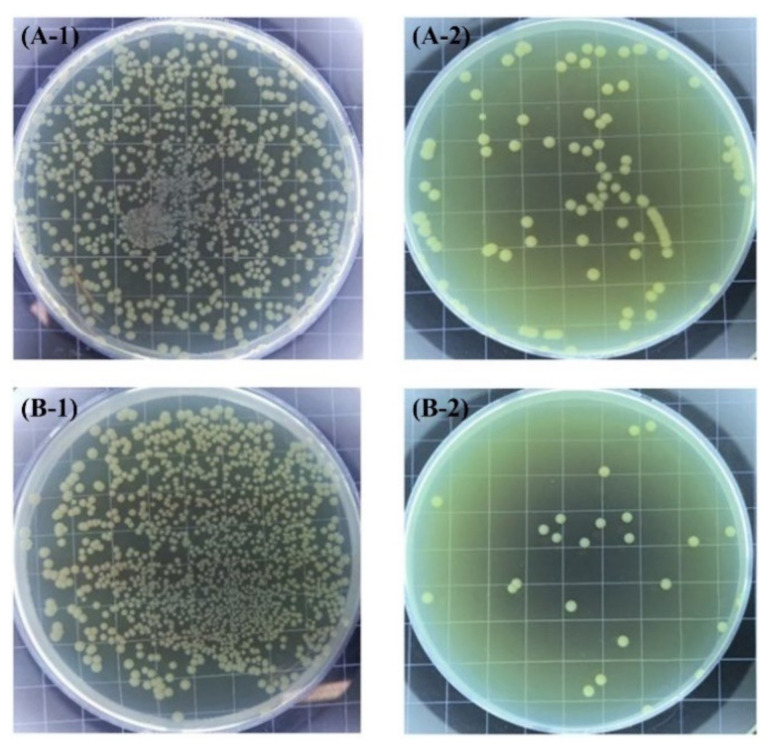
(**A**) Anti-*S. aureus;* and (**B**) anti-*E. coli* assay of untreated (−1) and copper nanoparticles (Cu NPs) (−2) surfaces. Figure derived from the work of Cao et al. (Reproduced from [86], published by John Wiley and Sons, 2019.).

The inhibitory mechanism of copper nanoparticles is still not entirely clear. Tsai et al. [98] suggest that the formation of stable copper-protein complexes causes interference in the transport of essential elements and causes oxidative stress, generating different cellular dysfunctions, such as the suppression of cell division and an increase in membrane permeability. Cao et al. [86] suggest that Cu^2+^ ions in water destroy the phospholipid layer of the cell wall of gram-negative bacteria. It was stated that Cu^2+^ ions can trigger the release of intracellular organic compounds (cell permeability), destroying the cell integrity of algae. The antifouling effect of the copper nanoparticles observed by Cao et al., on *E. coli* and *S. aureus* bacteria can be observed in Figure 6.

It was also suggested that the transport of Cu^2+^ ions into the cell is facilitated in gram-negative bacteria due to the presence of “porins” [99], which makes the interaction more pronounced. Porins are transmembrane proteins in gram-negative bacteria which form channels for the passage of molecules or particles of equal or smaller size than proteins [99]. In gram-positive bacteria, the thickness of the peptidoglycan layer and the lack of porins makes the penetration of Cu^2+^ ions slower and more complex [86].

Ghesemian et al. [85], stated that the antibacterial effect of copper nanoparticles is mainly due to the surface area available for the copper-bacteria interaction, that is, the size of the particle. When particles have more surface area available to interact with bacteria, their antibacterial effect tends to increase, and they become more cytotoxic to microorganisms [10,11,12]. The authors were able to synthesize nanoparticles of 8 nm, inhibiting the growth of the bacteria *Listeria monocytogenes* (67%) and *Pseudomonas aeruginosa* (74%) on both steel and glass surfaces. A 32 mg/L minimum concentration of nanoparticles is required to inhibit *Pseudomonas aeruginosa* and 16 mg/L to inhibit *Listeria monocytogenes*. The authors also emphasized that the inhibition is greater on glass surfaces than on steel, which can be explained by the high hydrophobicity of the bacteria. The binding of microorganisms to surfaces is often controlled by such interactions [100,101]. In addition to hydrophobicity, the adhesion of bacteria on different surfaces depends on the surface charge and the properties of electron donors and acceptors [49,102]. 

**Zinc**. Abi Nassif et al. [87] evaluated calcium alginate and zinc nanoparticle coatings on 316L stainless steel surfaces. They achieved good bacterial inhibition with: *Halomonas aquamarine*, *Vibrio aesturianus*, *Pseudoalteromonas elyakovii* (between 50 and 70%), and algae *Halomphora coffeaeformis* and *Cylindrotheca closterium* (between 70 and 90%).

The antibacterial and anti-algae activity of alginate with Cu^2+^ and Zn^2+^ ions was described by [103] who explained that these ions tend to form strong bonds with the thiol or sulfhydryl (−SH), imidazole (C_3_H_4_N_2_), amino (−NH_2_) and carboxyl (−COOH) groups of the membrane proteins of microorganisms. Structural changes in the membrane (increased permeability) mean that microorganisms are unable to properly regulate the transport of essential elements, leading to cell death.

**Silver**. With a broad spectrum of microbial activity, there are concerns about the toxicity of silver to mammalian cells and other non-target organisms [104]. Silver nanoparticles can reach bacterial cell walls, causing loss of membrane integrity and cell lysis [105,106].

**Figure 7:**
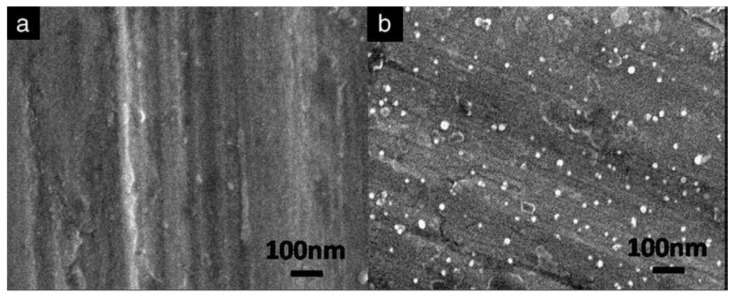
SEM images of the samples: (**a**) blank stainless steel sheet; and (**b**) AgNPs-stainless steel sheet obtained by Chen et al. (Reproduced from [90], published by Elsevier, 2010.).

Chen et al. [90] suggest that when stainless steel is exposed to water, it produces a large amount of hydroxyl (−OH) groups on the surface. When immersing the steel in 3-aminopropyltriethoxysilane (APTES), a layer of it is attached to the steel surface by Si−O−Cr covalent bonding by dehydrating the Si−OH and Cr_2_O_3_−OH bonds in the steel. In addition, the amino group (−NH_2_) can coordinate with the silver atoms, thus allowing a firm binding of the silver nanoparticles to the stainless steel surface, as shown in Figure 7 [107]. The antibacterial activity shown in this study was over 90%. The inhibition mechanism suggests that when Ag^+^ ions encounter bacteria they interact with the sulphur, nitrogen, or phosphorus atoms in the membrane, inhibiting growth and even killing the bacteria. 

**Figure 8:**
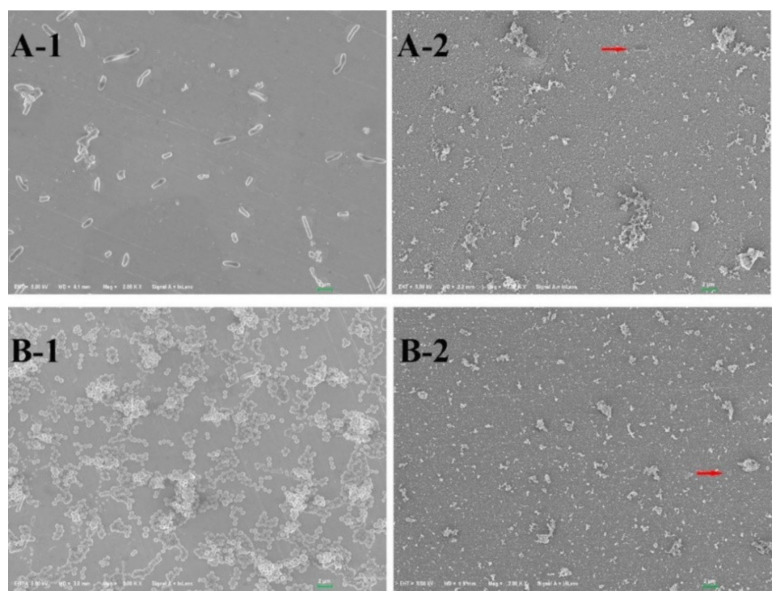
SEM images of: *E. coli* (**A**); and *S. aureus* (**B**) adhered on the surfaces of untreated samples (**A-1** and **B-1**) and DA-SS AgNPs (**B-2** and **B-2**). According to the work of (Reproduced from [91], published by Elsevier, 2018.).

Cao et al. [91] succeeded in depositing in situ silver nanoparticles on AISI 304 SS stainless steel by applying a polydopamine based in a weak alkaline solution and AgNO_3_. To synthesize these nanoparticles, the catechol groups of dopamine are commonly used. These active groups are oxidized in a weak alkaline solution to form intermediate groups called quinone methylates. Adhesive crosslinking is generated by a reverse dismutation reaction between the catechol or o-quinone groups of polydopamine and other compounds related to the catechol group. The quinone structures and catechol groups of oxidized dopamine act as a reducing agent for Ag^+^ ions in a AgNO_3_ solution. Subsequently, Ag^0^ binds to the nitrogen and oxygen sites in the polydopamine layer. Catechol groups of dopamine were oxidized to form intermediate groups (quinone methides), an inverse dismutation reaction between the catechol or o-quinone groups of polydopamine and other compounds related to catechol contributed to the formation of an adhesive reticular base on a stainless steel surface. Quinone structures and catechol groups of the oxidized dopamine acted as a chelating agent and reduction site for the reduction of the Ag^+^ ion when the reticulated surface was immersed in an AgNO_3_ solution. Later the Ag^0^ joined with the N and O atoms present in the polydopamine layer and grew. The modified surfaces showed an inhibition capacity of 99.9% for *Escherichia coli* bacteria and 99.5% for *Staphylococcus aureus* bacteria (see Figure 8). In addition, the surface modification produced an inhibitory capacity on the algae *Chlorela pyranoidosa*, *Phaeodctylum tricornutum* and *Naviculaceae* spp., of 98.5%, 98.2% and 98.7%, respectively, in relation to the untreated steels. According to these authors, the antibacterial activity of these nanoparticles is associated with direct damage to the cell wall by the imminent contact. As for the inhibitory mechanism in algae, they suggest that the Ag^+^ ion released on the surface of the nanoparticles interacts with the algae, triggering the release of proteins and polysaccharides in the algal cells, thus killing them, or inhibiting their growth [108,109]. An advantage of this method over others is that the efficiency did not change significantly after four weeks of immersing the samples in algae-containing solutions. This indicates that the behaviour of the modified nanoparticles is stable and durable.

Krishnan et al. [44] showed the antibacterial effect of silver nanoparticles on 15 different bacteria. They found that the maximum inhibition was for *Escherichia coli* (71.9%) and the minimum inhibition was for the bacterium *Micrococcus* sp. (40%). Regarding the inhibitory mechanism, Thiel et al. [110] suggest that nanoparticles bind to thiol or sulfhydryl groups of enzymes and proteins in the cell membrane, thus affecting protein biosynthesis and consequently the DNA and RNA of bacterial cells.

Feng et al. [111] demonstrated that Ag^+^ ions interact with thiol groups of proteins and DNA bases, leading to a respiratory inhibition of bacteria or unwinding of DNA, resulting in bacterial death or inhibition.

Regarding the mortality rate of marine biofouling fauna, Krishnan et al. [44] observed that with a minimum concentration of 50 μg/mL of nanoparticles, a mortality rate of 13.3% in the crustacean *Artemia* sp. was recorded at 24 h, while with a concentration of 200 μg/mL, the mortality recorded was 43.3%. In 48 h, the mortality rate doubled. On the other hand, the test on barnacle settlement showed a dependence of nanoparticle concentrations on the inhibition of larval settlement. A concentration of 250 μg/mL silver nanoparticles showed a 100% mortality rate in only 12 h, results that show the potential of silver nanoparticles as an antifouling agent.

**Anatase (TiO_2_)**. This broad-spectrum bactericide has excellent biocompatibility and corrosion resistance [112,113]. TiO_2_ particles are photoreactive and can kill or inhibit bacterial growth through cell wall penetration. 

The term “photoreactive” indicates that, with a suitable light source, these particles are activated, generating electrons and spaces that react with adsorbed water or molecular oxygen, producing reactive oxygen species (ROS). These species, in turn, interact with bacterial cells, killing them or inhibiting their growth [114,115]. Among the reactive species produced, −OH radicals stand out. These can destroy the bacterial cell wall by breaking covalent bonds and inhibiting the formation of crosslinks in the peptidoglycan layer, which is mainly responsible for the stability of the cell wall in bacteria [116,117]. The latter affects the reproduction function and the ability of microorganisms to infect the host tissue for a short period [117,118]. The degradation will depend on the duration of the illumination and the pH of the medium.

Zhang et al. [88] produced a TiO_2_/polytetrafluoroethylene (PTFE) coating on 316L stainless steel which exhibited antimicrobial and anticorrosive properties in body fluids. 

Polytetrafluoroethylene (PTFE) is a biomaterial of low surface energy that in a metal matrix significantly reduces the Lifshitz–van der Waals apolar component of coatings. This is one of the reasons why composite coatings have antibacterial properties [119,120]. These authors evaluated bacterial adhesion and growth of *Escherichia coli* and *Staphylococcus aureus*. They found that a surface coated thus had the least bacterial adhesion compared to individual TiO_2_ and PTFE coatings. They were able to reduce the adherence of *Escherichia coli* by 70.9% and *Staphylococcus aureus* by 65% after 24 h compared to the uncoated 316L stainless steel surface. To achieve these properties, the authors first formed a polydopamine layer to improve adhesion and homogeneity, and then deposited a TiO_2_/PTFE composite layer on top of the polydopamine layer. This coating showed the advantage of biocompatibility with mouse fibroblast cells, making it a potential alternative for protecting devices used in healthcare.

In simulated body fluids, Zhang et al. [88] showed that the TiO_2_/PTFE coating had the highest open circuit potential value, indicating higher thermodynamic stability. This coating had the best substrate protection as it significantly decreased the corrosion current (I_corr_) of the coated steel with respect to that of uncoated steel. Their results also showed that the TiO_2_/PTFE combination in the coatings improved the corrosion resistance compared to individual TiO_2_ and PTFE coatings.

Li et al. [121] reported that TiO_2_ can eliminate both gram-negative and gram-positive bacteria, due to the tendency of reactive oxygen species (such as −OH ions) to attack the peptidoglycan layer. 

**Figure 9:**
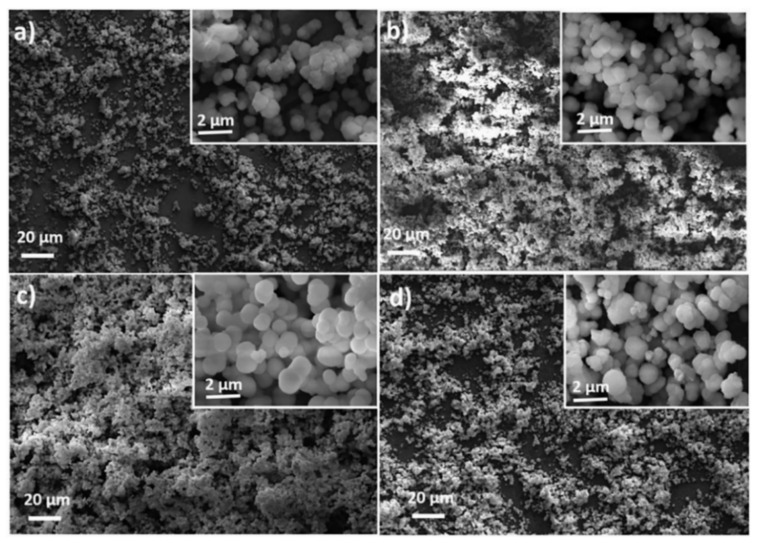
SEM micrographs obtained by Lopes et al. (Reproduced from [89], published by Elsevier, 2017.) of: (**a**) stainless steel; (**b**) DLC; (**c**) TiO_2_-DLC (0.1 g/L); and (**d**) TiO_2_-DLC (0.3 g/L) surfaces after biomineralization.

Lopes et al. [89] developed a novel coating based on crystalline apatite, diamond-like carbon and TiO_2_ nanoparticles grown on 304 stainless steel, shown in Figure 9. They evaluated the antibacterial effect of these coatings against *Staphylococcus aureus* bacteria. The hydroxyapatite/TiO_2_-DLC film at a concentration of 0.3 g/L showed a significant difference in the reduction of bacterial colonies. This finding was related to the decrease of the contact angle of the diamond-like carbon (DLC) films to such an extent that it was not possible to measure the contact angle after mineralization (θ = 0°). Furthermore, they observed that the action of hydroxyapatite as a synergistic agent enhanced the antimicrobial function of TiO_2_.

**Figure 10:**
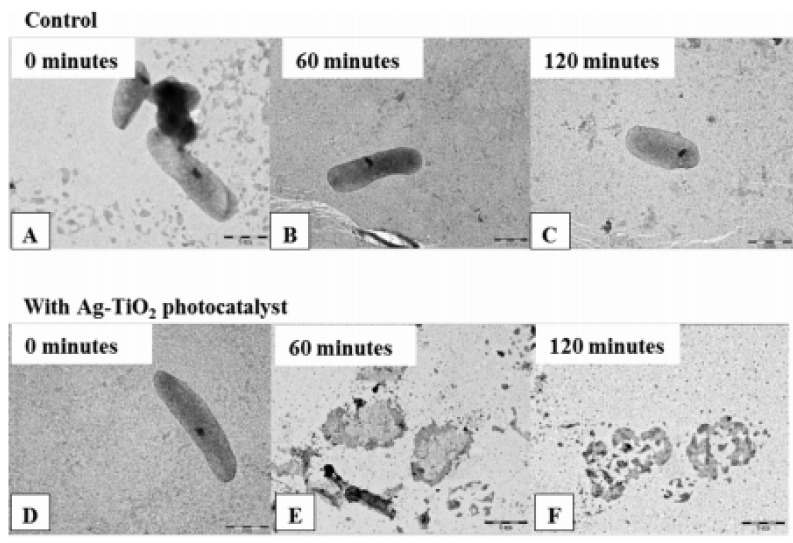
Representative TEM images of the sacculi of *E. coli*: (**A**) before reaction; (**B**) after 60 min; and (**C**) 120 min of only visible light photocatalysis under illumination with LED-light source (λ = 405 nm) as control measurements; and (**D**) after 0; (**E**) 60; and (**F**) 120 min on Ag–TiO_2_ photocatalyst under the same conditions. Figure derived from the work of Tallósy et al. (Reproduced from [13], published by Elsevier, 2016.).

Tallósy et al. [13] proposed that the wavelength that causes the photo-reactive effect in TiO_2_ nanoparticles can be extended to the visible region by adding silver nanoparticles without releasing a significant amount of Ag^+^ ions into the environment. They developed polyacrylate-based surface coatings that could be activated under visible light, inhibiting *Pseudomonas aeruginosa*, *Staphylococcus aureus* and *Escherichia coli* bacteria by 45.54%, 61.35% and 75.68%, respectively. They also observed the degradation of peptidoglycan and the outer membrane of bacterium cells by photocatalysis in the studied bacteria. Finally, the addition of silver nanoparticles gave a 15% improvement in bactericidal activity compared to pure TiO_2_ coatings. These results indicate that the use of TiO_2_−Ag coatings in marine structures could be very useful. Among the highlights of their work, the authors showed the photocatalytic operation of the coating on *E. coli*, as seen in Figure 10.

### 4.2. Generation of Nanotextures on Stainless Steel

Bacterial adhesion and growth mechanisms are highly dependent on the topography and chemistry of the stainless steel surface, and the interaction of bacteria with nanotextured surfaces will vary depending on the type of microorganism. Gram-negative bacteria that have a more fluid outer membrane will behave differently than the rigid peptidoglycan coating of gram-positive bacteria on nanotextured surfaces [122].

**Figure 11:**
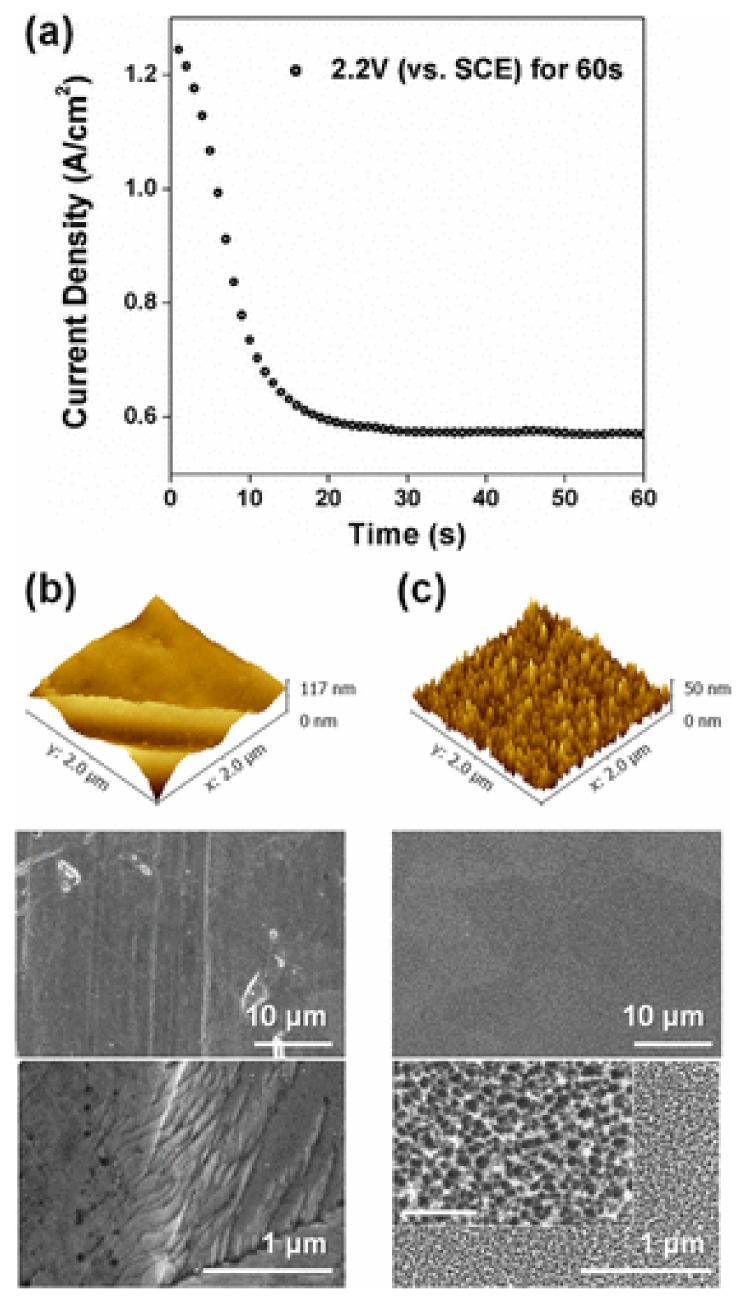
Structure characterization of stainless steel 316L surfaces after electrochemical modification: (**a**) potentiostatic polarization at an anodic potential of 2.2 V (vs. a saturated calomel electrode (SCE)) for fabricating a nanotextured SS316L (Nanotexturized-SS316L) surface. Three-dimensional AFM topography profiles and SEM images of: (**b**) as received (AR-SS316L); and (**c**) nanotextured (NT-SS316L) surfaces. The scale bar of the inset SEM image is 200 nm. Figure derived from the work of Jang et al. (Reproduced from [92], published by ACS, 2018, further permission related to the material excerpted should be directed to the ACS.).

Jang et al. [92] observed that the surface of 316L stainless steel nanotextured by electrochemical pickling reduced adhesion by up to 86.2% of *Staphylococcus aureus*, and up to 99.6% of *Escherichia coli* after 48 h of immersion in their respective culture media. The antibacterial mechanism of the nanotexture is attributed to the formation of controlled nanopores and nanoprotrusions with a diameter of 20 nm (see Figure 11). The sharp edges can induce mechanical stress on the membrane of adherent bacteria, resulting in cell death without the application of antibiotics, metallic, or polymeric coatings [122]. Competition between cell membrane elasticity and the capillarity of nanopores on the surface of steels can also improve the deformation and tension of the bacterial membranes [92].

The generation of nanotextures on stainless steel requires further experimentation to be considered as an antifouling alternative, as it has not yet been verified as effective in natural environments where several external factors may play a role. These factors include weather, turbulent flow, particles suspended in the fluid, or the adhesion of larger organisms that could cause change in texture, eliminating the beneficial effect that nanotextures imparts in a laboratory environment. 

To test the effectiveness of nanotexturing, Choi et al. [123] found that an increase in surface roughness from nanometres to microns does not significantly reduce bacterial adhesion on 316L stainless steel compared to untreated 316L stainless steel. 

## 5. Nanomaterials with Potential Use to Inhibit Biofouling on Stainless Steel

The approaches described in Section 4 are those specifically reported to mitigate the biofilm phenomenon, and subsequent biofouling and microbiologically induced corrosion, on stainless steels. However, it is important to consider other alternatives that have been reported for the surfaces of different materials that could be used on stainless steel. Some works that show promise for marine environments, but that could hardly be used in food processing and in the human body, are described below.

Several materials with good biocidal and antifouling activity have been reported [14,15,124,125,126,127,128,129,130]. Among these, the following stand out: halloysite clay nanotubes, mesoporous silica nanocapsules, and layered double hydroxides. These nanomaterials showed their efficiency in marine environments on target species with low toxicity on non-target species, when accompanied by biocidal agents such as DCOIT (4,5-Dichloro-2-octyl- 3-isothiazolone), zinc and copper pyrithiones, and silver nanoparticles. These materials are called smart nano-containers, they are nanostructured materials that release the active compounds in a controlled way, avoiding contact between the active species and the coating matrix, providing a protective barrier [131]. The antifouling mechanisms of these nanomaterials are briefly explained below.

### 5.1. Mesoporous Silica Nanocapsules

**Figure 12:**
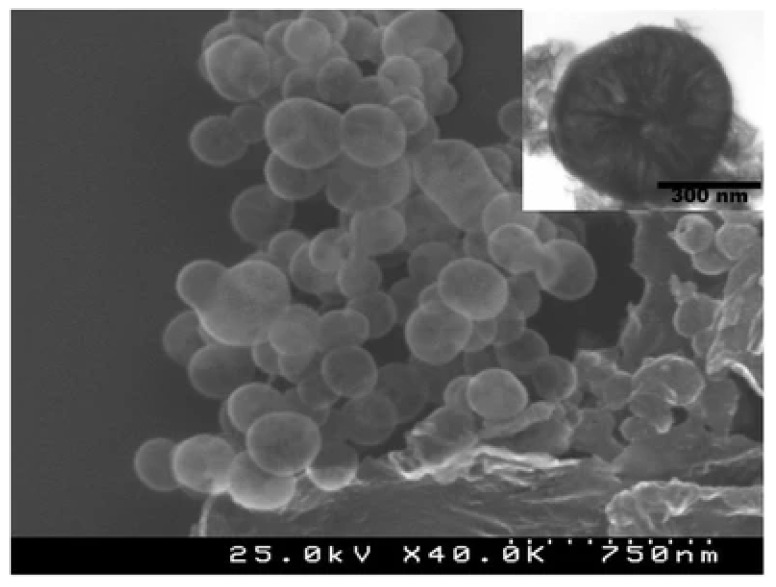
SEM image of mesoporous silica capsules. Inset shows the TEM image of an individual SiNC. Figure derived from the work of Avelelas et al. (Reproduced from [14], published by Springer Nature, 2017.).

These spherical nanoparticles typically have a diameter of 100–200 nm and the predominant release mechanism is based on the diffusion of the active compound through the porous layer [132,133]. The nanocapsules are prepared using an oil-in-water microemulsion, followed by hydrolysis, then condensation of the silica precursor (TEOS) at the interface of the microemulsion [133]. The precipitate obtained is filtered and washed with deionized water. The encapsulation of the biocide (zinc or copper pyrithione, as well as the DCOIT) is prepared similarly. The selected biocide was previously dissolved in the dispersed phase solution before the water/oil emulsion. Avelelas et al. [14], showed SEM and TEM images of mesoporous silicananocapsules (see Figure 12).

**Unloaded silica nanocapsules**. Unloaded silica nanocapsules inhibited the growth of the alga *P. tricornutum* [14]. Gutner-Hoch et al. [15] observed a mild toxic effect on the nauplii of the crustacean *Artemia salina* and the sea urchin *Paracentrotus lividus*. This can be attributed to the presence, within its porous structure, of a quaternary ammonium compound (CTAB Hexadecyltrimethylammonium bromide), which is used as an emulsion stabilizer during the synthesis of nanocapsules [130,133]. This compound was catalogued as a toxic compound for a wide range of aquatic organisms [134].

**Figure 13:**
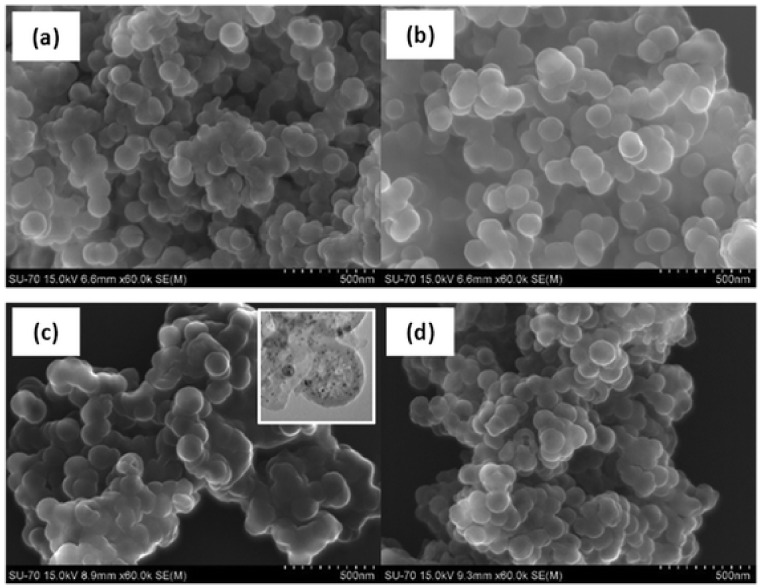
SEM images of: (**a**) empty SiNCs; (**b**) SiNCs with encapsulated DCOIT; (**c**) SiNCs coated with silver; and (**d**) SiNCs with encapsulated DCOIT and coated with silver. Inset: TEM image of SiNCs containing immobilized Ag nanoparticles on the surface. Figure derived from the work of Figueiredo et al. (Reproduced from [127], published by Royal Society of Chemistry, 2020.).

**Silica nanocapsules loaded with biocide.** The copper pyrithione-filled nanocapsules were very found to be effective against diatom growth; however, acute toxicity was observed for non-target species [128]. Gutner-Hoch [15] found that zinc pyrithiones are more toxic than those of copper for the sea urchin *Paracentrotus lividus*. The release of zinc and copper pyrithione is limited by the low solubility of biocides in seawater [135,136]. When applying a DCOIT biocide load, Santos et al. [130] observed that in gametes of the bivalve *P*. *perna,* the application of nanocapsules loaded with DCOIT was 137 times less toxic than free DCOIT. This substantial difference is related to the controlled release of the biocide that occurs gradually over time, and by predefined stimuli. The reduction of the hazard of free DCOIT in nanocapsules was also detailed by Figueiredo et al. [128]. The risk of free silver was also reduced by encapsulating it in the mesoporous nanocapsule, or by encapsulating both the silver and DCOIT. The lower risk of these new nanomaterials can be explained by their slow biocide release over time and their behavior in seawater, in particular their aggregation/agglomeration over time, and by their variable exposure concentration [128]. Figueiredo et al. [127] observed that both DCOIT and silver encapsulated in silica nanocapsules reduced their toxicity for eight non-target species (microalgae: *Isochrysis galbana* and *Nannochloropsis gaditana*; the rotifer: *Brachionus plicatilis*; the bivalve: *Cerastoderma edule*; Polychaetes: *Hediste diversicolor*; Crustaceans: *Artemia salina* and *Echinoderm: Paracentrotus lividus*) while showing good antifouling performance against three target species (bacteria: *A. fischeri*; diatom: *P. tricornutum* and mussel *M. galloprovincialis*). Figure 13 shows SEM images of the silica nanocapsules used in this study.

The toxicity of the encapsulated biocides was lower for all the species evaluated, and the toxicity of free DCOIT was 214 times higher than encapsulated DCOIT. The toxicity of the encapsulates may be due to physical/mechanical effects on certain species, such as [127,133,137,138,139]:

- Particles can adhere to gills, interfering with filtration/respiration, leading to sublethal effects that ultimately cause the death;
- Particles can adhere to the body surface, affecting mobility which can cause starvation and death;
- Particles can cause a shadowing effect on the microalgae, which can interfere with the photosynthesis process.

### 5.2. Layered Double Hydroxides

These plates typically have a thickness of 20–40 nm, and the release mechanism of the main active compound is anion exchange [132]. The synthesis of zinc and aluminum hydroxides is by the coprecipitation of metal hydroxide salts in a solution with excess sodium nitrate, where the pH range is adjusted with NaOH [140]. The obtained suspension is washed with deionized water and filtered under reduced pressure.

The encapsulation of zinc and copper pyrithione biocides is achieved by anionic exchange between nitrates (from the hydroxide structure) and the anionic form of pyrithione, by constant stirring, at room temperature [140]. Subsequently, the incorporated biocide suspension is filtered and washed with deionized water.

**Unloaded layered double hydroxides.** According to Avelelas et al. [14], when applied alone, double layer hydroxides have low toxicity for target species (such as *P. Tricornutum* and the mollusk *Mytilus edulis*) as against non-target species (such as algae *Tetraselmis chuii*). A similar observation was found by Gutner-Hoch et al. [129], who observed that the double layer hydroxides without biocidal loads were not toxic to the crustacean *Artemia salina* and to the sea urchin *Paracentrotus lividus*. Gutner-Hoch et al. [129] also observed that hydroxides without biocidal loading have a more pronounced antifouling effect on bryozoan larvae than on adult mussels.

**Layered double hydroxides loaded with biocide**. Results of the application of double layer hydroxides loaded with zinc pyrithione are promising, as they require a lower amount of biocide than that used with other compounds [14]. When these hydroxides are loaded with zinc and copper pyrithiones, there are more limitations with respect to silica nanocapsules due to the possibility of having a significant amount of pyrithione molecules chemically bound to the surface of the hydroxide. In the case of nanocapsules, pyrithiones are only physically trapped and can diffuse more easily, despite having a lower charge content [14].

Zinc pyrithione immobilized in double hydroxides was shown to have lower toxicity to algae (*P. tricornutum* and *T. chuii*) compared to pyrithiones encapsulated in silica nanocapsules, or with copper pyrithione alone. The double layer hydroxide with zinc pyrithione proved to be even more effective in terms of antifouling properties, showing a higher acute toxicity against the mussel *M. edulis* (macro-fouling species) compared to free zinc pyrithione [14]. Gutner-Hoch et al. [15] observed that pyrithiones encapsulated in double layer hydroxides showed less toxicity on the crustacean *Artemia salina* and the sea urchin *Paracentrutus lividus* compared to free pyrithiones. Gutner-Hoch et al. [15] observed that hydroxides loaded with pyrithiones are the most effective antimacroincrustants for mussels and bryozoans, with those loaded with zinc pyrithione being more effective for mussels, and those loaded with copper pyrithione being more effective for bryozoans.

Gutner-Hoch et al. [15], observed that the presence of zinc hydroxide-pyrithione can contribute to the formation and release of ionized pyrithione and Zn^2+^ ion. Although intracellular zinc levels can pose risks to the cell, pyrithione ions are highly reactive and tend to react with metals and generate new compounds, thus presenting a more toxic chemical mixture [14]. Pyrithiones are known to be powerful inhibitors of various cellular processes, such as membrane transport, regulation of ATP levels, and protein synthesis [141].

### 5.3. Halloysite Nanotubes

Halloysite is a naturally occurring mineral, widely available at low cost. It is a tubular material of rolled layers of aluminosilicates with an outer diameter of 50 to 60 nm, a lumen of 10 to 15 nm, and a length of 0.5 to 1 m [126]. It is like kaolinite in composition, with more water between the layers adjacent to the walls [126]. The outer surface of negatively charged silica and the lower surface of positively charged alumina in halloysite allow for the selective charging of chemicals [142,143].

These nanotubes can be used as containers for the encapsulation and controlled release of antifouling active agents. Sustained release can even be achieved for years. These inexpensive nanotubes can be used to contain antifouling agents such as DCOIT and serve as a template for the formation of silver particles, preventing their undesirable aggregation [144,145].

**Figure 14:**
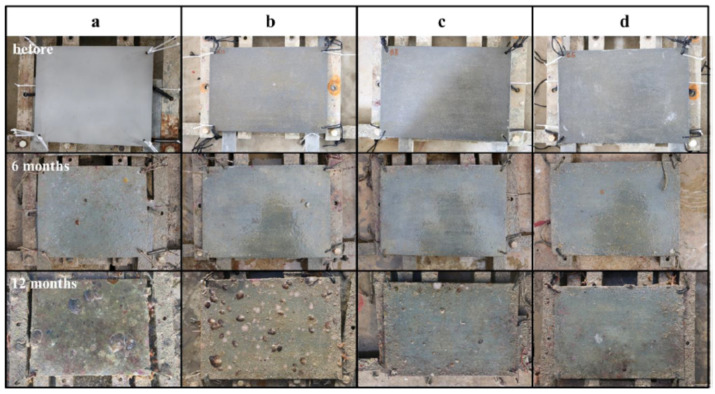
Photographs of the flat panels: (**a**) uncoated; (**b**) coated with epoxy resin directly doped with 5 wt.% DCOIT; (**c**) halloysite epoxy resin composites with 5 wt.% DCOIT plus 2 wt.% loaded in nanotubes; and (**d**) plus 5 wt.% loaded in nanotubes. Upper images—before; and lower images—after shallow submergence in Sanya Bay, South China Sea, March 2018–February 2019. Figure derived from the work of Fu et al. [125].

**Figure 15:**
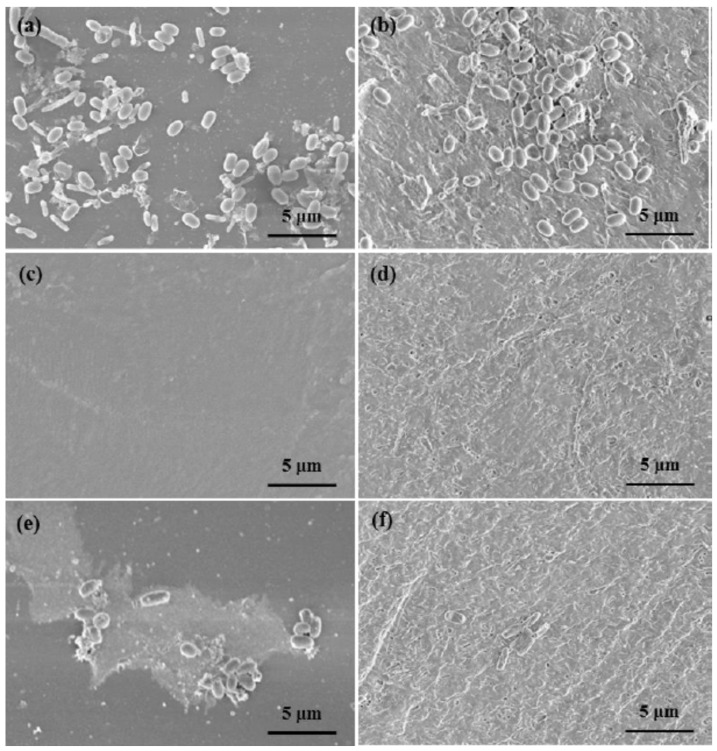
SEM images of the bacteria on the coating surface (left column—epoxy resin; right column—epoxy-halloysite formulations): (**a**) pure epoxy resin; (**c**,**e**) epoxy resin directly doped with DCOIT; (**b**) epoxy resin composited with pristine/empty halloysite; (**d**,**f**) epoxy resin with DCOIT-loaded halloysite. The samples were incubated in *Vibrio natriegens* suspension for three days (**c**,**d**) and after exposure to seawater for 60 days on a shaking platform. Figure derived from the work of Fu et al. [125].

Fu et al. [125], developed an epoxy antifouling coating doped with halloysite nanotubes loaded with the biocide (DCOIT) or silver, which provided prolonged protection against the proliferation of marine microorganisms. The epoxy-halloysite-DCOIT encapsulation extended its antifouling performance to 12 months (see Figure 14), with much less adhesion and proliferation of the marine bacterium *Vibrio natriegens* on the surface of the resin. Figure 15 shows the antifouling action of epoxy-DCOIT-hallysite coatings over a period of 60 days. They found that replacing 2% weight of DCOIT for halloysite in the traditional formula dramatically improved the antifouling properties of the epoxy coating. These findings suggest promise in scalability for these marine protective coatings. The researchers also found that the antibacterial property of epoxy resin can be enhanced with the addition of silver nanoparticles in the halloysite. Incorporating silver nanoparticles in the internal lumen of halloysite extends the stability of silver and the useful life of the antimicrobial effect. The antimicrobial activities of Ag-halloysite compounds depend on the amount of silver ions released: 10 mg/mL Ag-halloysite nanocomposites release approximately 1 mM Ag^+^ ions to the aqueous medium.

### 5.4. Surface Functionalization

The functionalization of surfaces, especially silica nanocapsules with some quaternary ammonium salts, conferred the possibility of having passive protection due to covalent bonding to the nanoparticle surface [124]. Quaternary ammonium salts are positively charged cationic compounds; bacterial cell membranes are negatively charged. Because of electrostatic interactions, QAS can adsorb on cell surfaces and diffuse through cell walls. Later it binds to the cytoplasmic membrane, which causes it to release K^+^ ions and essential components, leading to cell death. Meanwhile, the progressive release of the biocide has a passive protective effect [146]. It is well known that quaternary ammonium salts are toxic and put non-target species at risk. To solve this, more friendly replacements have been sought [124,147], with good, but not yet friendly enough, results.

## 6. Conclusions

To control the biofouling phenomenon, the critical stage is the formation of the biofilm on the substrate surfaces: this must be prevented. Three approaches have been analysed: 1. The use of metallic nanoparticles in an organic matrix; 2. The use of organic nanocomposite coatings; and 3. inhibiting the colonization of microorganisms, mainly bacteria and algae.

In the case of the marine environment, silver nanoparticles are reported to be the most effective in stainless steel, due to the silver-protein interaction and the available reactive surface of the nanoparticles. 

In the case of food processing, organic polymers, such as polyethylene glycol coatings, have shown good efficacy on stainless steel and pose the least risk for the consumer as they have good biocompatibility, are non-toxic, and modify only the hydrophilic character of the surface, causing slippage effects for the microorganism. 

In the case of devices used in health care, TiO_2_ nanoparticles associated with hydroxyapatite are preferred due to their anti-corrosion and antifouling characteristics, and excellent biocompatibility with the host organism. 

The bacteria most studied in the three environments are *Staphylococcus aureus* and *Escherichia coli*. It is considered that the analysis of these bacteria can serve as a model to inhibit the adhesion of gram-positive and gram-negative bacteria through experiments with new technologies, and by defining the selectivity of nanotechnology for each type of bacteria. Antifouling mechanisms include the metal-cell nanoparticle interaction (either: metal-protein or metal-functional group), which increases the permeability of the membrane of the microorganism. This causes the loss of essential compounds from the cell of the microorganism, annihilating it or inhibiting its development. This mechanism is most easily observed in gram-negative bacteria because the porins facilitate the transport of metal into the cell. The development of reactive oxygen species (ROS) has a more marked effect on gram-positive bacteria, due to their effectiveness in attacking the peptidoglycan layer, which is thicker than gram-negative bacteria. 

In marine environments and food processing, the generation of nano surface textures requires further experimentation. However, it has been demonstrated that they do have antifouling potential, based on the generation of mechanical stress in microorganisms due to the presence of peaks and pores on the modified surface. Some antibacterial agents used in biomedical applications may have potential in food processing or in the marine environment, due to the similarity of microorganisms found.

Mesoporous silica nanocapsules, layered double hydroxides, halloysite nanotubes, and surface functionalization can increase antifouling activity times by up to one year through the controlled release of biocides. Controlled release can also reduce the toxicity of biocides relative to their application in free form.

The application of nanotechnology has been proven effective as an antifouling agent at the laboratory level and shows a promising future for use in situ. However, there is still much work to be done to analyse performance under real operating conditions, as well as the application of any of the technologies evaluated in the three environments mentioned. Better antifouling strategies could be designed based on the knowledge of the mechanism of biofouling, and the mechanisms of the nanotechnologies analysed here in the short, medium, and long term. This would consolidate efforts to lengthen the lifetime of devices operating in these environments.

## Figures and Tables

**Table 1:** Macrofouling species.

Macro Fouling Species	Description	Reference
**Crustacean** *Balanus amphitrite* *Semibalanus balanoides* *Balanus improvises*	Barnacles are common and are particularly difficult to remove. They respond in complex ways to a variety of signals, particularly the quorum signals of their species. *Balanus improvisus* tends to prefer hydrophobic metal surfaces and relatively low flow velocities. *Balanus amphitrite* prefers hydrophilic surfaces and prefers to settle on surfaces exposed to flows of medium velocity.	[71,72,73,74]
**Bryozoa** *Bugula simplex* *Bugula stolonifera* *Bugula turrita* *Bugula neritina* *Bugula flabellata*	They tend to settle on substrates with mature biofilms; however, biofilms inhibit the settlement of *Bugula flabellata*.	[75,76,77]
**Tunicates or urochordates** *Diplosoma listerianum* *Didemnum candidum* *Ascidia mentula* *Ciona intestinalis*	Settlement of some tunicates is facilitated or attracted by biofilms and generally increases as biofilms age.	[76]
**Cnidarians** *Clava multicornis* *Porites astreoides* *Balanophyllia elegans* *Alcyonium siderium* *Dynamena pumila*	Their ideal place of settlement is often in algae already settled on the material, where there is less sediment, in the bottom part of the algae.	[78,79,80]
**Annelids** *Spirorbis spirorbis* *Spirorbis tridentatus* *Pomatoceros lamarckii* *Hydroides elegans*	They tend to settle in large amounts on the surface of the material at low tide, once the biofilm has dried.	[78]
**Sponges or beads** *Reneira* *Cliona celata*	Sponge larvae do not feed. They are ephemeral and have limited dispersal. Since sponges lack adhesive glands, to adhere to a surface their ectodermal cells secrete adhesive.	[81]
**Molluscs** *Dreissena polymorpha* *Mytilus edulis*	Molluscs, unlike other organisms, are able to metamorphose in other places and move towards the material. The blue mussel, *Mytilus edulis*, prefers rough biofilms and hydrophobic surfaces for settlement.	[82]

Names highlighted in bolt for species identification only.

**Table 2:** Microorganisms that have shown reduction or inhibition of adhesion/growth on stainless steel surfaces because of nanotechnology treatment.

Type of Stainless Steel	Inhibited Bacteria	Inhibited Algae	Suggested Treatment	Work Environment	Reference
Not specified	*GR + Staphylacococcus aureus* *GR − Escherichia coli*	-	Vanadium pentoxide (V_2_O_5_) particles	Marine	[84]
Not specified	*GR + Listeria monocytogenes* *GR − Pseudomonas aeruginosa*	-	Copper particles	Food and medicine	[85]
SS 304	*GR + Staphylacococcus aureus* *GR − Escherichia coli*	*Phaeodactylum triconutum*	Copper particles (Polidopamide matrix)	Marine	[86]
SS 316L	*GR − Halomonas aquamarina* *GR − Vibro aesturianus* *GR - Pseudoalteromonas elyakovii*	*Halamphora coffeaeformis Cylindrotheca closterium*	Zinc particles (Calcium alginate matrix)	Marine	[87]
Not specified	*GR − Pseudomonas aeruginosa* *GR + Staphylocuccus aureus* *GR − Escherichia coli*	-	Anatase particles (TiO_2_ − Ag)	Medicine	[13]
SS 316L	*GR − Escherichia coli* *GR + Staphylococcus aureus*	-	TiO_2_-PTFE (Polytetrafluoroethylene)	Medicine	[88]
SS 304	*GR + Staphylocuccus aureus*	-	TiO_2_-CID (Diamond-like carbon)	Medicine	[89]
SS 304	*GR − Escherichia coli*	-	Ag-APTES (3-aminopropyl triethoxysilane)	Food	[90]
SS 304	*GR − Escherichia coli* *GR + Staphylococcus aureus*	*Chlorella pyrenoidosa Phaeodactylum tricornutum Naviculaceae* spp.	Silver nanoparticles in a polidopamine matrix	Marine	[91]
SS 304	*GR − Escherichia coli* *GR − Flavobacterium* sp. *GR − Pseudomonas aeruginosa* *GR − Aeromonas* sp. *GR − Vibrio cholerae* *GR − Salmonella* sp. *GR − Shigella* sp. *GR − Enterobacter aerogenes* *GR − Klebsiella* sp. *GR − Chromohalobacter* *GR + Bacillus* sp. *GR + Micrococcus* sp. *GR + Corynebacterium* sp. *GR + Bacillus litoralis* *GR + Staphylococcus aureus*	-	*Silver particles—algae turbine ornate*	Marine	[44]
SS 316L	*GR + Staphylococcus aureus* *GR − Escherichia coli*	-	Generation of nanotextures	Food and medicine	[92]

## Data Availability

As this is a review article, the data supporting the results can be found in the respective references in the manuscript.
