# Correction: Pérez et al. Use of Nanotechnology to Mitigate Biofouling in Stainless Steel Devices Used in Food Processing, Healthcare, and Marine Environments. *Toxics* 2022, *10*, 35

**DOI:** 10.3390/toxics10050229

**Published:** 2022-04-29

**Authors:** Hugo Pérez, Gregorio Vargas, Rodolfo Silva

**Affiliations:** 1 Sustentabilidad de los Recursos Naturales y Energía, Centro de Investigación y de Estudios Avanzados del Instituto Politécnico Nacional (CINVESTAV) Unidad Saltillo, Ramos Arizpe 25900, Mexico; hugo.perez@cinvestav.mx; 2 Instituto de Ingeniería, Universidad Nacional Autónoma de México, Mexico City 04510, Mexico; rsilvac@iingen.unam.mx

The authors would like to make corrections to their published paper [1]. The changes are as follows:

In Section 3, “The Biofouling Process”, Figure 3, with the title “The biofouling process on substrate surfaces in the sea”, should be deleted. This figure was removed to avoid a possible copyright conflict with some of the images that were integrated into Figure 3.

The order from Figure 4 to Figure 16 will be reduced by one, and the callouts in the main text should be modified accordingly.

The sentence referring to Figure 3 in the manuscript “Figure 3 shows the four stages in the biofouling process on seawater devices” should change to “The stages in the biofouling process in seawater devices are described below”.

The authors apologize for any inconvenience caused and state that the scientific conclusions are unaffected. The original article has been updated.
